# Comparisons of the Sexual Cycles for the Coccidian Parasites *Eimeria* and *Toxoplasma*

**DOI:** 10.3389/fcimb.2020.604897

**Published:** 2020-12-14

**Authors:** Bruno Martorelli Di Genova, Laura J. Knoll

**Affiliations:** Department of Medical Microbiology and Immunology, University of Wisconsin-Madison, Madison, WI, United States

**Keywords:** coccidia, *Toxoplasma*, *Eimeria*, sexual reproduction, merozoite, schizont

## Abstract

*Toxoplasma gondii* and *Eimeria* spp. are widely prevalent Coccidian parasites that undergo sexual reproduction during their life cycle. *T. gondii* can infect any warm-blooded animal in its asexual cycle; however, its sexual cycle is restricted to felines. *Eimeria* spp. are usually restricted to one host species, and their whole life cycle is completed within this same host. The literature reviewed in this article comprises the recent findings regarding the unique biology of the sexual development of *T. gondii* and *Eimeria* spp. The molecular basis of sex in these pathogens has been significantly unraveled by new findings in parasite differentiation along with transcriptional analysis of *T. gondii* and *Eimeria* spp. pre-sexual and sexual stages. Focusing on the metabolic networks, analysis of these transcriptome datasets shows enrichment for several different metabolic pathways. Transcripts for glycolysis enzymes are consistently more abundant in *T. gondii* cat infection stages than the asexual tachyzoite stage and *Eimeria* spp. merozoite and gamete stages compared to sporozoites. Recent breakthroughs in host-pathogen interaction and host restriction have significantly expanded the understating of the unique biology of these pathogens. This review aims to critically explore advances in the sexual cycle of Coccidia parasites with the ultimate goal of comparing and analyzing the sexual cycle of *Eimeria* spp. and *T. gondii*.

## Introduction

Coccidia is a subclass of the phylum Apicomplexa that includes a wide range of obligatory intracellular parasites. Coccidia pathogens were first described in 1879 by Leuckart as ovoid cells found in a patient sample (Dobell, 1922). Many global pathogens, like *Toxoplasma gondii* and *Eimeria* spp., belong to the Coccidia (Robben and Sibley, 2004). All species in the phylum Apicomplexa, including the subclass Coccidia, reproduce sexually (Smith et al., 2002; Walker et al., 2013). As obligatory intracellular pathogens, Coccidia must infect and parasitize a host to complete their life cycle (Entzeroth et al., 1998; Gupta et al., 2012). The host range is determined by the possible different species that a pathogen can parasitize (Reid et al., 2012), giving species-specific parasites a limited host range. The host range of most *Eimeria* spp. is limited to one single species, whereas only the sexual cycle of *Toxoplasma gondii* has a narrow host range only including species of the family Felidae (Kogut, 1990).

Even though all Coccidia have a sexual cycle, the most molecular characterization has been performed on *T. gondii* and *Eimeria* spp.; therefore, they are the focus of this review. Pioneering work has also been done in *Hammondia*, *Besnoitia*, *Neospora*, and *Sarcocystis* (García-Lunar et al., 2014; Blazejewski et al., 2015; Sokol et al., 2018; Horcajo et al., 2018; García-Sánchez et al., 2019; Jiménez-Meléndez et al., 2020) and further molecular characterization of these parasites will be fundamental for further understanding of sexual regulation and commitment in Coccidia. In this review, we highlight the most recent findings regarding sexual development for *T. gondii* and *Eimeria* spp. For example, the differences in transcript expression between sexual and asexual stages revealed many stage-specific genes in *T. gondii* and *E. tenella* (Walker et al., 2015; Hehl et al., 2015; Su et al., 2017). During merogony and sexual differentiation, the expression of genes responsible for fundamental cellular processes, such as metabolism and host-pathogen interaction, changes widely (Behnke et al., 2014; Reid et al., 2014; Walker et al., 2015; Hehl et al., 2015; Su et al., 2017; Ramakrishnan et al., 2019). In this review, these differences will be explored and correlated to additional discoveries in the sexual cycles of *T. gondii* and *Eimeria* spp.

## Toxoplasma and Eimeria Life Cycles

Both *T. gondii* and *Eimeria* spp. undergo sexual reproduction with a restricted host range during their life cycle (Kogut, 1990; Reid et al., 2012). Despite the host restriction in the sexual cycle, *Eimeria* spp. and *T. gondii* have fundamental differences in their life cycles. The genus *Eimeria* contains around 1,700 described species (Walker et al., 2013; Clark et al., 2017). In general, *Eimeria* spp. are monoxenous parasites, as they complete their life cycles in a single host (Barta, 1989; Walker et al., 2013; Clark et al., 2017). The genus *Toxoplasma* only contains one species, *T. gondii*, and its sexual cycle is restricted to felines (Martorelli Di Genova et al., 2019). For its asexual cycle, *T. gondii* can infect many warm-blooded animals, including several species of mammals and birds. In the intermediate host, *T. gondii* replicates asexually and differentiates into persistent tissue cysts, containing the stage called bradyzoites (Ong et al., 2011).

**Figure 1:**
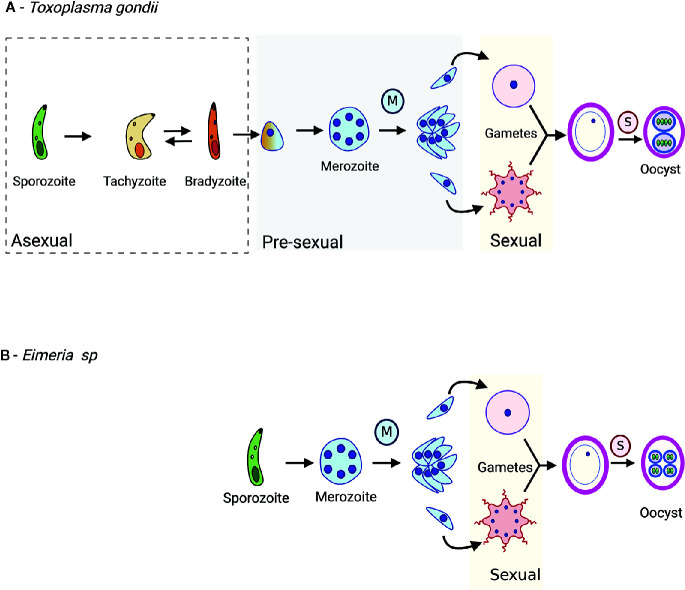
The life cycle of *T. gondii* **(A)** and *Eimeria* spp. **(B)**. After host cell invasion, *T. gondii* bradyzoites may either differentiate into a pre-sexual stage or a tachyzoite. *T. gondii* tachyzoites are a fast-replicative stage and disseminate the infection in the host. *Eimeria* sporozoites replicate asexually and differentiate into pre-sexual stages. The pre-sexual stages for *T. gondii* and *Eimeria* spp. are schizonts and merozoites because they precede the sexual stages and are committed to sexual development. During merogony (blue M), nuclei replication form multi-nucleated cells, schizonts, that post cytoplasmic division, generates merozoites. Merozoites can either keep replicating or differentiate into sexual stages. The two gametes, macrogamete, and microgamete fuse and generate oocyst. Sporulation (pink S) occurs in the early oocyst, generating sporozoites. Lastly, *T. gondii* sporozoite differentiates into tachyzoites after an oocyst infects the host.

*T. gondii* is classified as a cyst forming Coccidia because it develops into tissue cysts in the intermediate hosts during the asexual life cycle (Dubey, 2020). This asexual cycle can be completed successively, as these tissue cysts can be passed indefinitely within intermediate hosts. After ingestion, *T. gondii* bradyzoites can either differentiate into a tachyzoite to start the asexual cycle or a pre-sexual stage to start the sexual cycle, depending on whether the host is a non-feline or feline, respectively (Martorelli Di Genova et al., 2019). However, that is not the case for *Eimeria* spp. (**Figure 1**). *Eimeria* spp. do not form tissue cysts and do not have intermediate hosts because their whole life cycle is complete within the same host (Lal et al., 2009; Walker et al., 2013). *Eimeria* spp. sporozoites always differentiate into pre-sexual stages *in vivo* (Walker et al., 2013; Walker et al., 2015).

The life cycle of *Eimeria* spp. starts when a new host ingests oocysts by fecal-oral contact or through contaminated food or water (Robben and Sibley, 2004). In the case of *T. gondii*, the sexual cycle usually starts upon the ingestion of tissue cysts containing bradyzoites (Martorelli Di Genova et al., 2019). There is experimental evidence that *T. gondii* tachyzoites or oocysts can start the sexual cycle in cats; however, oocyst shedding is significantly delayed (Dubey, 2005). The prepatent period is 3–8 days when cats are fed bradyzoite cysts, but 5–34 days when cats are fed tachyzoites and 18–41 days when cats are fed oocysts. This delay likely indicates that additional developmental steps occur before the tachyzoites or sporozoites can undergo sexual development, perhaps even becoming transitioning through the bradyzoite stage. For both *T. gondii* and *Eimeria* spp., the oocyst or cyst wall is digested in the stomach, and the parasites are liberated to infect the intestine (Russell and Sinden, 1981). While *T. gondii* sexual development exclusively occurs in the feline small intestine (Martorelli Di Genova et al., 2019), different *Eimeria* species have distinct host tissue tropism for their sexual cycles, such as the liver or gallbladder, but most occur within the intestinal tract (Dubey, 1986; Walker et al., 2013; Walker et al., 2015; Sivajothi et al., 2016).

In both *T. gondii* and *Eimeria* spp., merozoites can either continue replicating or differentiate into sexual stages (**Figure 1**) (Su et al., 2017). For Eimeria spp., the number of rounds of merozoite replication is genetically determined and differs between the species and isolates and can respond to artificial selection (Montes et al., 1998; Pakandl, 2005). There are two distinct sexual stages: macrogamete and microgamete. Macrogametes, or female gametes, remain intracellular while the microgametes, or male gametes, can swim in the extracellular environment *via* flagellum until they invade a new cell (Walker et al., 2015). Upon the invasion, fusion of microgamete with macrogamete forms a diploid zygote (**Figure 1**), followed by the formation of a protective wall, resulting in an unsporulated oocyst (Walker et al., 2013). The newly formed oocysts are shed within the host feces (Bussière et al., 2018). Once released into the environment, oocysts may sporulate depending on the conditions (Zhou et al., 2016). During oocyst sporulation, the diploid zygote replicates by meiosis, generating haploid sporozoites (**Figure 1**) (Striepen et al., 2007; Dubey, 2019).

## Host Specificity

The extensive speciation in *Eimeria* is remarkable, as this genus contains around 1,700 described species (Clark et al., 2017). Most described *Eimeria* spp. are thought to be restricted to a single host species; however, experimental evidence suggests that some *Eimeria* spp. that infect rodents have a broader host range (Clark et al., 2017; Mácová et al., 2018; Jarquín-Díaz et al., 2020). The biological mechanisms underlying *Eimeria* speciation and host restriction are unknown. Two independent studies did not find evidence of coevolution between *Eimeria* spp. and their respective host species (Kvičerová and Hypša, 2013; Vrba and Pakandl, 2015). One of these studies suggests that *Eimeria* specification is likely caused by the adaptation of the parasite to its host, rather than a cophylogenetic process (Kvičerová and Hypša, 2013).

The sexual cycle, but not the asexual cycle of *T. gondii*, presents a host range restricted to felines (Dubey, 2020). Felines are exclusive carnivores and are auxotrophic for both taurine and desaturated fatty acids with more than two double bonds, such as arachidonic acid (Sinclair et al., 1979; Rentschler et al., 1986). Feline arachidonic acid auxotrophy is due to the lack of delta-6-desaturase (D6D) activity in their intestines (Sinclair et al., 1979). The absence of this enzyme activity is not observed in other mammals. D6D adds additional double bonds to fatty acids, and it is fundamental for arachidonic acid synthesis (Obukowicz et al., 1998). This phenotype results in the systemic accumulation of linoleic acid (LA) in felines (Sinclair et al., 1979). Our group showed that high levels of LA are required for *T. gondii* bradyzoites to differentiate into pre-sexual stages (Martorelli Di Genova et al., 2019). We showed that supplementation of mice diet with LA and a D6D inhibitor is enough to promote the sexual cycle of *T. gondii* in a rodent, breaking the species barrier (Martorelli Di Genova et al., 2019). It remains unknown the reason why LA is crucial for *T. gondii* sexual development. In fungi, LA has been shown to interfere with *Aspergillus* and *Ophiostoma* cellular development (Calvo et al., 1999; Naruzawa et al., 2015). In these fungal species, oxylipins derived from LA were characterized as signaling molecules with important roles in the fungi sexual and asexual differentiation (Naruzawa et al., 2015; Fischer and Keller, 2016). These findings suggest the possibility of LA, or derivatives molecules, having signaling roles during *T*. *gondii* sexual development.

Pre-sexual stages for both *Eimeria* spp. and *T. gondii* replicate in a process known as merogony (Ramakrishnan et al., 2017; Ma et al., 2019). During merogony, parasite cells replicate their nuclei at a high rate (Dubey et al., 2017) forming cells with many nuclei sharing the same cytoplasm (Sheffield, 1970; Dubey, 2017). Merozoites are developed by the end of this process, and they can differentiate into gametes after replicating asexually (Ferguson and Dubremetz, 2020). The replication rate by which the genetic material and the parasites themselves replicate is presumed to be higher for pre-sexual stages than any other Coccidia life stage (Hehl et al., 2015).

## Transcriptomic Analysis of Metabolic Pathways

**Figure 2:**
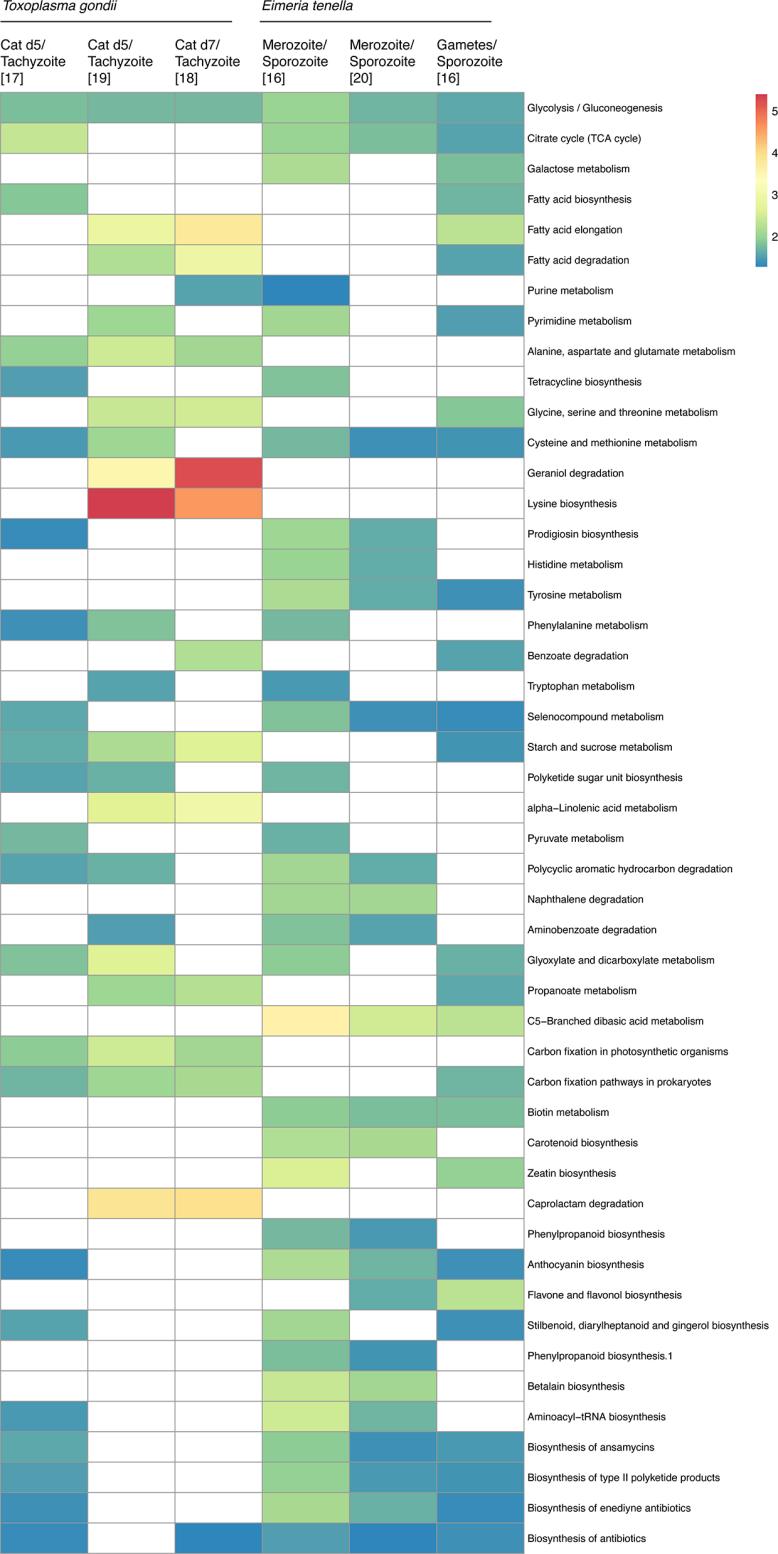
Metabolic pathway enrichment from transcriptional analysis of pre-sexual and sexual stages. Pre-sexual stage transcriptomics was compared using KEGG pathway analysis on ToxoDB (Gajria et al., 2008). The heatmap shows how many times each metabolic pathway is enriched in pre-sexual stages compared to asexual stages for each dataset. Pathways that were not enriched are marked as white. All pathways presented were enriched in at least two independent datasets. Figure created using ClustVis (Metsalu and Vilo, 2015). Tg, *Toxoplasma gondii*; Et, *Eimeria tenella.* For *T. gondii* sexual stages, only sample EES5 was used for the comparison.

Independent transcriptomes of *E. tenella* and *T. gondii* have shown the upregulation of a significant number of metabolic pathways in the pre-sexual stages compared to other developmental stages (Behnke et al., 2014; Reid et al., 2014; Walker et al., 2015; Hehl et al., 2015; Ramakrishnan et al., 2019). Analysis of the transcriptome datasets for *E. tenella* and *T. gondii* (Gajria et al., 2008) shows enrichment for different metabolic pathways (**Figure 2**). The datasets used in this analysis were obtained from published data (Behnke et al., 2014; Reid et al., 2014; Walker et al., 2015; Hehl et al., 2015; Ramakrishnan et al., 2019) and were all previously normalized. We analyzed only the three datasets available for *T. gondii* cat infection stages that were simultaneously compared to tachyzoites, as well as two independent studies of *E. tenella* merozoite and gamete stages compared to sporozoites. Apart from the variability observed among the datasets, there are important trends shared between the different experiments.

Many metabolic pathways are enriched across the independent datasets. Central carbon, especially glycolysis, and amino acid metabolism pathways are enriched among all datasets for the pre-sexual stages of both organisms. These findings are corroborated by a proteomics study of *E. tenella* that reported the overexpression of several metabolic enzymes associated with oxidative phosphorylation in the pre-sexual stages compared to other life stages (Lal et al., 2009). Amino acid pathways are also enriched in pre-sexual and sexual stages (Behnke et al., 2014; Reid et al., 2014; Walker et al., 2015; Hehl et al., 2015; Ramakrishnan et al., 2019). Apart from *E. tenella*, transcriptomics of other poultry infecting species *Eimeria maxima* and *Eimeria necatrix* as well as the mouse infecting species *Eimeria falciformis* also suggested higher metabolic needs in pre-sexual and sexual stages compared to sporozoites (Su et al., 2017; Ehret et al., 2017; Hu et al., 2018), highlighting an overall trend of higher metabolic demand in the pre-sexual stages of Eimeria spp. For *Eimeria bovis*, pre-sexual stage replication *in vitro* is directly impacted by host cellular sterol profile, demonstrating the intrinsic parasite dependency on the host metabolism and nutrient availability (Taubert et al., 2018). The variability between pre-sexual stages datasets might be a consequence of the multiple differentiation steps that precede sexual differentiation. Also, it appears that the same pathways upregulated in the pre-sexual stages are still upregulated in sexual stages (**Figure 2**). Because it is not possible yet to purify *T. gondii* gametes or merozoites, the data sets from these samples likely had a combination of both, so it is unclear how similar the metabolism between pre-sexual and sexual stages is. An overall similarity was previously observed between *T. gondii*, *E. tenella*, and *E. falciformis* pre-sexual and sexual stages transcripts expressions, further supporting our analysis (Ehret et al., 2017).

It is known that *T. gondii* tachyzoites usurp and modify host metabolism to fulfill their metabolic needs and replicate intracellularly (Krishnan et al., 2020; Olson et al., 2020). It has been hypothesized that the massive replication during merogony has a higher energy demand than tachyzoite replication (Behnke et al., 2014; Hehl et al., 2015). Additionally, critical metabolites and metabolic pathways could be required for the sexual cycle of these parasites. This hypothesis is corroborated by the specific requirement of LA, but not oleic acid, for sexual differentiation of *T. gondii* (Martorelli Di Genova et al., 2019). In higher eukaryotes, it has been shown that differences in metabolism are responsible for cellular differentiation (McGraw and Mittal, 2010; Gatie and Kelly, 2018). Therefore, the overall metabolic upregulation could be intrinsically related to the parasite differentiation during pre-sexual development and not simply a consequence of accelerated replication. It is fundamental to highlight that transcriptomic data is not a measure of metabolite abundance, and direct analysis of metabolites is needed to characterize pre-sexual and sexual stage metabolism fully.

## Pre-Sexual Stages

Apart from metabolic pathways, many other cellular functions are likely altered in the pre-sexual stages. Transcriptional analysis of *T. gondii* pre-sexual stages reveals changes in the expression of transcripts critical for host-pathogen interaction (Behnke et al., 2014; Hehl et al., 2015; Ramakrishnan et al., 2019). Independent transcriptomes showed that pre-sexual and sexual stages downregulate the expression of most rhoptry and granular protein genes that are upregulated in tachyzoites (Hehl et al., 2015; Ramakrishnan et al., 2019). Similarly, the expression of rhoptry kinases is higher in sporozoites than pre-sexual stages for *E. falciformis* (Heitlinger et al., 2014). In *T. gondii* tachyzoites, rhoptry proteins have important known functions, including the biogenesis of the moving junction, which is required for invasion (Dlugonska, 2008; Pernas and Boothroyd, 2010). Some rhoptry proteins are secreted into the host cell and can associate with host mitochondria, endoplasmic reticulum, or be translocated to the host nucleus (Dlugonska, 2008; Pernas and Boothroyd, 2010). Some rhoptry proteins are also involved in the biogenesis of the parasitophorous vacuole, while dense granule proteins are generally responsible for its architecture and function (Pernas and Boothroyd, 2010; Gold et al., 2015).

For *T. gondii*, only two pre-sexual stage effector proteins have been characterized, TgGRA11B and TgBRP1. TgGRA11B is exclusively expressed in *T. gondii* pre-sexual stages but changes its localization from the dense granules to the parasitophorous vacuole membrane as the pre-sexual stages develop (Hehl et al., 2015; Ramakrishnan et al., 2017). *T. gondii* pre-sexual stages also express TgBRP1, a known bradyzoite rhoptry protein (Schwarz et al., 2005). The role of TgBRP1 and TgGRA11B is still unclear during pre-sexual development, but these findings show that the pre-sexual forms have unique interactions with the host compared to the asexual stages of infection. A transcriptomic analysis also showed that *T. gondii* pre-sexual stages overexpress different surface antigens linked to GPI, further suggesting differences in its interaction with the host (Hehl et al., 2015). *E. tenella* also expresses pre-sexual stage surface antigens. These stages overexpress many surface antigens (SAG) linked to glycosylphosphatidylinositol (GPI) in comparison with sporozoites (Reid et al., 2014). Another study using these *E. tenella* pre-sexual overexpressed SAGs showed that some of them suppressed expression of interferon-gamma and IL-12 in macrophages *in vitro* and induced expression of IL-10 (Chow et al., 2011). Another subset of these SAGs induced nitric oxide production in macrophages, potentially being related to the inflammatory response caused by *E. tenella* infection (Chow et al., 2011).

Another important side of host-pathogen interaction during pre-sexual development is the host immune response to the infection. Transcriptomic analysis of *T. gondii* infected cats showed a high expression of transcripts crucial for the immune response (Cong et al., 2018). Examples of these transcripts with high expression in infected cats include interferon-gamma and different cytokines (Cong et al., 2018). Most reports describe that toxoplasmosis infection causes mild symptoms in immunocompetent felines, suggesting that the immune system can clear the infection or at least cause the parasites to differentiate into the chronic infection bradyzoite stage (Calero-Bernal and Gennari, 2019). Host features may vary the *T. gondii* oocyst shedding rates, including cat breed as a potential factor (Must et al., 2017).

In contrast to *T. gondii* infection in cats, the *Eimeria* spp. infection often harms the host. The infection by *Eimeria* spp. typically results in malabsorption, consequential weight loss, and mortality in-floor raised chickens (Quiroz-Castañeda and Dantán-González, 2015). Increased intestinal IL-10 expression during *Eimeria* spp. infection in chickens is suggested to be related to the symptoms (Arendt et al., 2019). On the other hand, interferon-gamma seems protective against avian coccidiosis, as shown in many studies (Kim et al., 2019).

Limited tissue culture options are one major roadblock for studying pre-sexual and sexual stages and their interaction with the host. Since the 1960s, efforts have been made to maintain Coccidia *in vitro* (Strout et al., 1965). A recent finding shows that chicken epithelial cell line supports *E. tenella* sexual development, from sporozoites to gametes (Bussière et al., 2018). For *T. gondii*, LA supplemented feline or D6D-inhibited mouse organoid support pre-sexual and sexual stages development, but without infectious oocyst production (Martorelli Di Genova et al., 2019).

## Sexual Commitment and Differentiation in Coccidia

Commitment to sexual differentiation is a key process during the life cycle of Apicomplexa parasites. After commitment, the parasite developmental program will culminate with its differentiation into a sexual stage (Bechtsi and Waters, 2017). For example, sexual commitment in the Apicomplexa parasite *Plasmodium falciparum* is a well-described process. The sexual commitment of *P. falciparum* parasites relies on the expression of a specific ApiAP2 factor, named PfApiAP2-g. ApiAP2s are transcriptional factors found in Apicomplexa that have been shown to regulate virulence and cellular development in these parasites (Jeninga et al., 2019). The name derives from the apetala transcription factors 2 (AP2), a family of transcription factors discovered in plants (Jeninga et al., 2019). Single-cell transcriptomics of PfApiAP2-G conditional depleted parasites revealed that this factor upregulates the expression of other transcription factors and histone modifiers, and defined PfApiAP2-G as a master regulator of gametogenesis (Poran et al., 2017). The expression of PfApiAP2-G is regulated by histone methylation and acetylation, demonstrating a critical epigenetic regulation of the parasite differentiation (Bechtsi and Waters, 2017).

Previous studies have described that *T. gondii* has 67 ApiAP2 factors, and many of them are stage-specific according to transcriptomic studies (Hehl et al., 2015; Kim et al., 2020). Seminal work showed that the differentiation of *T. gondii* tachyzoites into bradyzoites is tightly regulated by multiple ApiAp2, demonstrating a central role of these transcription factors in this parasite’s development (Radke et al., 2013; Hong et al., 2017). The microrchidia protein (MORC) in a complex with ApiAP2 factors is responsible for repressing sexual commitment in tachyzoites (Farhat et al., 2020). The complex MORC-ApiAP2 is responsible for recruiting a histone deacetylase, TgHDAC3, that promotes specifically the deacetylation of bradyzoite and pre-sexual and sexual stage-specific genes, further disrupting their expression (Farhat et al., 2020). The addition of a histone deacetylase inhibitor, FR235222, to tachyzoites induces their differentiation into bradyzoites in fibroblasts in tissue culture (Bougdour et al., 2009). These findings suggest that epigenetics might play a critical role in *T. gondii* differentiation, as observed in the sexual commitment of *P. falciparum* (Bechtsi and Waters, 2017).

The differentiation of *T. gondii* intro bradyzoites is orchestrated by a Myb-like transcription factor named BFD1 (Waldman et al., 2020). BFD1 induced bradyzoite differentiation is independent of a change in the media pH or any other cellular stress (Mayoral et al., 2020). In the life cycle of *T. gondii*, when a feline consumes an intermediate host, the bradyzoite is the developmental stage that precedes the pre-sexual ones. It would be relevant to explore if BFD1 and MORC together regulate bradyzoite development and commitment to the sexual cycle.

The number of ApiAP2 factors varies from 44 to 54 in *Eimeria* spp. infecting chickens (Reid et al., 2014), while the mouse infecting species E. *falciformis* contains only 17 genes containing an AP2 domain (Ehret et al., 2017). As observed in *T. gondii*, cellular differentiation is likely regulated by ApiAP2 factors in *Eimeria* spp. Transcriptional analysis of the pre-sexual stages of *E. tenella* revealed that ApiAP2 expression profile changes during pre-sexual development (Su et al., 2017). Individual deletion of 10 out of 33 ApiAP2 factors was successful in a screening performed in sporozoites of *E. tenella* (Hu et al., 2020). This finding indicates that these 10 ApiAP2 are likely dispensable in *E. tenella*. Lastly, sexual commitment in *Eimeria* spp. has been proposed to be genetically programmed and not reliant on environmental clues, as observed in *P. falciparum* and likely the case for *T. gondii* (Smith et al., 2002; Walker et al., 2013).

### Final Steps of the Sexual Cycle: Gametogenesis and Oocyst Biology

Transcriptomics of *E. tenella* showed upregulation of over 800 transcripts during gametogenesis compared to sporozoites, identifying many gamete-specific transcripts (Walker et al., 2015). Macrogamete specific transcripts are intrinsically related to oocyst wall biogenesis. According to this study, distinct wall protein transcripts and transcripts surface antigens are upregulated in macrogametes. Macrogametes also upregulate proteases, oxidoreductases, glycosylation, and fatty acid metabolism transcripts. This finding gives an initial insight into the mechanisms for the oocyst wall biosynthesis.

The same study shows that microgametes upregulate axonemes, flagella, DNA condensation, and transcripts likely related to gamete fusion (Walker et al., 2015). Additionally, a study using *E. falciformis* confirmed the trends observed for *E. tenella* gametes upregulated transcripts (Ehret et al., 2017). These putative transcripts might play a significant role in microgamete locomotion and fusion to the macrogamete. HAPLESS2 (HAP2) is a known microgamete protein in *P. falciparum* with a role in fertilization (Vega-Rodriguez et al., 2015). The *E. tenella* (Walker et al., 2015) and *T. gondii* (Ramakrishnan et al., 2019) orthologues are highly expressed during the sexual development of both species. The deletion of TgHAP2 results in non-infectious oocysts with aberrant morphology and generates a putative anti-*Toxoplasma* vaccine strain (Ramakrishnan et al., 2019). Gamete fusion and meiosis in *T. gondii* was indirectly demonstrated by crossing two single drug-resistant strains and obtaining haploid strains resistant to both drugs (Pfefferkorn and Pfefferkorn, 1980). Remarkable electron microscopy work showed *T. gondii* microgametes attached to a macrogamete or early oocysts, suggesting the occurrence of fertilization (Ferguson and Dubremetz, 2020). Fertilization in *E. maxima* appears to be affected by heat stress (Schneiders et al., 2020). In this study, heat-stressed parasites presented different transcriptional profile compared to the control; EmHAP2 transcript expression, for example, was significantly decreased (Schneiders et al., 2020). Another pioneering study showed that cross-fertilization is common in polyclonal infections of *E. tenella*, further demonstrating the occurrence of fertilization in this parasite (Blake et al., 2015).

After fertilization of gametes, oocysts form within the host cells and are subsequently shed within the host feces. Coccidia oocysts are resistant to environmental stress, surviving extreme conditions, such as UV (Ware et al., 2010), hypochlorite, and ozone (Wainwright et al., 2007). Oocysts remain viable, and likely infectious, for many months after shedding (Dubey, 2019). The structure and chemical composition of the oocyst wall is responsible for the oocyst resilience (Dumètre et al., 2013).

The oocyst wall is composed of two distinct structures: outer and inner walls (Ferguson and Dubremetz, 2020). The outer wall surrounds the oocyst, while the inner wall is physically attached to the outer wall and in contact with the cellular membrane (Mai et al., 2009; Bushkin et al., 2013). Despite the interaction between inner and outer walls, they are independent, as hypochlorite treatment strips off only the outer wall while the inner one remains intact. In *E. tenella* and *E. maxima*, proteins correspond to at least 90% of the outer wall composition weight, and lipids and carbohydrates are responsible for 10% of the weight, 8%, and 2%, respectively (Mai et al., 2009). Bushkin et al. showed that lipids present in the oocyst wall of *T. gondii* and *E. tenella* wall were a complex mixture of triglycerides, similar to *Mycobacterium* walls, and could be acid-fast stained (Bushkin et al., 2013).

One interesting transcript upregulated in *E. tenella* macrogametes is amiloride-sensitive amine oxidase (EtAO2). The protein coded by this gene contains an extracellular adhesive MAM (meprin, A5, μ domain) domain that likely plays a significant role in oocyst wall biogenesis. AO2 activity catalyzes the cross-linking of tyrosine rich peptides, forming dityrosine bonds that are involved in the oocyst wall hardening (Belli et al., 2003; Walker et al., 2015).

The presence of dityrosine bonds in the oocyst wall was first indicated by a seminal study using *E. maxima* as a model. This study identified tyrosine-rich glycoproteins, EmGAM56, and EmGAM82, and showed that they are proteolytically processed and incorporated in *E. maxima*’s oocyst wall (Belli et al., 2003). Dityrosine bonds are also present in the *T. gondii* oocyst wall (Wang Z. T. et al., 2017). *T. gondii* transcriptomics showed upregulation of TgAO2 and transcripts from tyrosine-rich glycoproteins orthologues in the late stages of sexual reproduction (Ramakrishnan et al., 2019). The expression of TgAO2 in macrogametes was demonstrated by immunofluorescence, suggesting a conserved mechanism for Coccidia wall biogenesis (Walker et al., 2015). The presence of dityrosine bonds in the oocyst wall of *Eimeria* and *T. gondii* confers the oocyst wall autofluorescent when excited with blue light (Belli et al., 2003; Ramakrishnan et al., 2019).

A subset of proteins named oocyst wall proteins (OWP) is transcriptionally expressed in both *E. tenella* and *T. gondii* (Walker et al., 2015; Ramakrishnan et al., 2019). Orthologs of these cysteine-rich OWPs were first characterized in *Cryptosporidium* (Templeton et al., 2004). There are 7 cysteine-rich OWPs in *T. gondii* (named OWP1-7), and they localize to the oocyst wall (Possenti et al., 2010). OWPs play a role in the oocyst wall structure and have a central role in the oocyst wall of many Apicomplexan (Templeton et al., 2004; Possenti et al., 2010). While no further characterization of the OWPs has occurred yet for *E. tenella*, one study showed that two OWPs are expressed during sporulation and present in the sporocyst wall in *Eimeria nieschulzi* (Jonscher et al., 2015) further suggesting additional roles for OWPs in Coccidia parasites.

## Concluding Remarks

Transcriptional studies of *Eimeria* spp. and *T. gondii* revealed many key components that are shared in their sexual cycle. The upregulated metabolism into the pre- and sexual stages may explain the complexity in reproducing these stages *in vitro*. A central hypothesis is that without the fulfilled metabolic needs, Coccidia differentiation in pre-sexual and sexual stages is reduced or completely ablated. This hypothesis is further suggested by the sexual cycle of *T. gondii*, relying on the LA concentration (Martorelli Di Genova et al., 2019). Along with lipid metabolism, other intestinal physiology factors might be critical to consider. The presence of bile salts, hypoxia, high osmotic pressure, and the microbiota are all intestinal conditions that need to be considered (Boyer, 2013; Guinane and Cotter, 2013). Primary cells and organoid models can support partial sexual development of Coccidia parasites, but infectious oocyst production has not yet been observed in cell culture (Bussière et al., 2018; Martorelli Di Genova et al., 2019). However, *in vitro* fertilization of *Cryptosporidium* gametes has occurred in some cell lines, highlighting that while it is challenging to reproduce the Apicomplexa life cycle in tissue culture, it is not impossible (Heo et al., 2018; Wilke et al., 2019). One significant difficulty is to obtain sporozoites from Coccidia oocysts and culture them. Recently improved techniques might significantly improve this procedure and maximize tissue culture yields (Sokol et al., 2020).

Although there are similarities between the sexual cycles of *T. gondii* and *Eimeria* spp., there are also substantial differences. Likely these life cycle differences result in unique sexual commitment, regulation, and development between the two Coccidia genera. Conversely, the *T. gondii* sexual cycle appears to be triggered by host factors. This hypothesis is corroborated by the presence of two distinct masters of *T. gondii* differentiation, BFD1 and MORC (Farhat et al., 2020; Waldman et al., 2020). Furthermore, epigenetics is likely to play a central role in *T. gondii* development, as exemplified by a study using histone deacetylase inhibitors and its effect on development (Bougdour et al., 2009). Additional studies are necessary to define further how *T. gondii* and *Eimeria* spp. parasites commit to the sexual cycle and whether each genus has a unique sexual regulation mechanism.

While sexual reproduction is a common trait in the phylum Apicomplexa (Smith et al., 2002), *T. gondii* and closely related organisms from the Sarcocystidae family have an independent asexual cycle. After these pathogens infect an intermediate host, they develop into tissue cysts. As tissue cysts are infectious for both intermediate and definitive host, Sarcocystidae parasites can infect intermediate hosts indefinitely, skipping the definitive host, and sexual reproduction as a result. It is unclear how long *T. gondii* strains replicate asexually in nature and what the evolutionary consequences are of continually missing sexual reproduction.

Chronic toxoplasmosis is still a morbidity factor for immunocompromised individuals (Wang Z.-D. et al., 2017). Both Coccidia pathogens put at risk the economy, especially regarding livestock and agriculture. Toxoplasmosis causes recurrent abortions in sheep (Shahbazi et al., 2019). Avian coccidiosis from Eimeria spp. is responsible for increases in weight loss and mortality in chickens resulting in significant economic losses for the poultry industry yearly (Noack et al., 2019). Overall, to study these pathogens, it is necessary to comprehend both basic biology concepts as well to generate medical and economic relevant knowledge.

## Author Contributions

BG wrote the review and generated the figures. LK edited the review and figures. All authors contributed to the article and approved the submitted version.

## Conflict of Interest

The authors declare that the research was conducted in the absence of any commercial or financial relationships that could be construed as a potential conflict of interest.

## References

[B1] Arendt M. Elissa J. Schmidt N. Michael E. Potter N. Cook M. (2019). Investigating the role of interleukin 10 on Eimeria intestinal pathogenesis in broiler chickens. Vet. Immunol. Immunopathol. 218, 109934. 10.1016/j.vetimm.2019.109934 31520870 PMC6861699

[B2] Barta J. R. (1989). Phylogenetic Analysis of the Class Sporozoea (Phylum Apicomplexa Levine, 1970): Evidence for the Independent Evolution of Heteroxenous Life Cycles. J. Parasitol. 75, 195–206. 10.2307/3282766 2494316

[B3] Bechtsi D. P. Waters A. P. (2017). Genomics and epigenetics of sexual commitment in Plasmodium. Singap. Malar. Netw. Meet. SingMalNet 47, 425–434. 10.1016/j.ijpara.2017.03.002 28455236

[B4] Behnke M. S. Zhang T. P. Dubey J. P. Sibley L. D. (2014). Toxoplasma gondii merozoite gene expression analysis with comparison to the life cycle discloses a unique expression state during enteric development. BMC Genomics 15, 350. 10.1186/1471-2164-15-350 24885521 PMC4035076

[B5] Belli S. I. Wallach M. G. Luxford C. Davies M. J. Smith N. C. (2003). Roles of tyrosine-rich precursor glycoproteins and dityrosine- and 3,4-dihydroxyphenylalanine-mediated protein cross-linking in development of the oocyst wall in the coccidian parasite Eimeria maxima. Eukaryot. Cell 2, 456–464. 10.1128/EC.2.3.456-464.2003 12796290 PMC161462

[B6] Blake D. P. Clark E. L. Macdonald S. E. Thenmozhi V. Kundu K. Garg R. (2015). Population, genetic, and antigenic diversity of the apicomplexan Eimeria tenella and their relevance to vaccine development. Proc. Natl. Acad. Sci. 112, E5343. 10.1073/pnas.1506468112 26354122 PMC4586875

[B7] Blazejewski T. Nursimulu N. Pszenny V. Dangoudoubiyam S. Namasivayam S. Chiasson M. A. (2015). Systems-Based Analysis of the Sarcocystis neurona Genome Identifies Pathways That Contribute to a Heteroxenous Life Cycle. mBio 6, e02445–e02414. 10.1128/mBio.02445-14 25670772 PMC4337577

[B8] Bougdour A. Maubon D. Baldacci P. Ortet P. Bastien O. Bouillon A. (2009). Drug inhibition of HDAC3 and epigenetic control of differentiation in Apicomplexa parasites. J. Exp. Med. 206, 953–966. 10.1084/jem.20082826 19349466 PMC2715132

[B9] Boyer J. L. (2013). Bile Formation and Secretion. Compr. Physiol. 3, 1035–1078. 10.1002/cphy.c120027 23897680 PMC4091928

[B10] Bushkin G. G. Motari E. Carpentieri A. Dubey J. P. Costello C. E. Robbins P. W. (2013). Evidence for a structural role for acid-fast lipids in oocyst walls of Cryptosporidium, Toxoplasma, and Eimeria. mBio 4, e00387–e00313. 10.1128/mBio.00387-13 PMC3760245 24003177

[B11] Bussière F. I. Niepceron A. Sausset A. Esnault E. Silvestre A. Walker R. A. (2018). Establishment of an in vitro chicken epithelial cell line model to investigate Eimeria tenella gamete development. Parasitol. Vectors 11, 44. 10.1186/s13071-018-2622-1 PMC5774133 29347990

[B12] Calero-Bernal R. Gennari S. M. (2019). Clinical Toxoplasmosis in Dogs and Cats: An Update. Front. Vet. Sci. 6, 54. 10.3389/fvets.2019.00054 30863754 PMC6399377

[B13] Calvo A. M. Hinze L. L. Gardner H. W. Keller N. P. (1999). Sporogenic effect of polyunsaturated fatty acids on development of Aspergillus spp. Appl. Environ. Microbiol. 65, 3668–3673. 10.1128/AEM.65.8.3668-3673.1999 10427064 PMC91549

[B14] Chow Y.-P. Wan K.-L. Blake D. P. Tomley F. Nathan S. (2011). Immunogenic Eimeria tenella glycosylphosphatidylinositol-anchored surface antigens (SAGs) induce inflammatory responses in avian macrophages. PLoS One 6, e25233–e25233. 10.1371/journal.pone.0025233 21980402 PMC3182191

[B15] Clark E. L. Tomley F. M. Blake D. P (2017). Are Eimeria genetically diverse, and does it matter? Trends Parasitol. 33, 231–241. 10.1016/j.pt.2016.08.007 27593338

[B16] Cong W. Dottorini T. Khan F. Emes R. D. Zhang F.-K. Zhou C.-X. (2018). Acute Toxoplasma Gondii Infection in Cats Induced Tissue-Specific Transcriptional Response Dominated by Immune Signatures. Front. Immunol. 9. 10.3389/fimmu.2018.02403 PMC6202952 30405608

[B17] Dlugonska H. (2008). Toxoplasma rhoptries: unique secretory organelles and source of promising vaccine proteins for immunoprevention of toxoplasmosis. J. Biomed. Biotechnol. 2008, 632424–632424. 10.1155/2008/632424 18670609 PMC2486357

[B18] Dobell C. (1922). The Discovery of the Coccidia. Parasitology 14, 342–348. 10.1017/S0031182000010258

[B19] Dubey R. Harrison B. Dangoudoubiyam S. Bandini G. Cheng K. Kosber A. (2017). Differential Roles for Inner Membrane Complex Proteins across Toxoplasma gondii and Sarcocystis neurona Development. mSphere 2, e00409–e00417. 10.1128/mSphere.00409-17 29062899 PMC5646244

[B20] Dubey J. (1986). Coccidiosis in the gallbladder of a goat. Proc. Helminthol. Soc Wash. 53, 277–281.

[B21] Dubey J. P. (2005). Unexpected oocyst shedding by cats fed Toxoplasma gondii tachyzoites: in vivo stage conversion and strain variation. Vet. Parasitol. 133, 289–298. 10.1016/j.vetpar.2005.06.007 16024176

[B22] Dubey J. P. (2017). Schizogony and gametogony of oocyst-deficient T-263 strain of Toxoplasma gondii. Vet. Parasitol. 245, 160–162. 10.1016/j.vetpar.2017.05.024 28624132

[B23] Dubey J. P. (2019). Coccidiosis in Livestock, Poultry, Companion Animals, and Humans. (Boca Raton, Florida: CRC Press).

[B24] Dubey J. P. (2020). “Chapter 1 - The history and life cycle of Toxoplasma gondii,” in Toxoplasma gondii (Third Edition). Eds. Weiss L. M. Kim K. (Academic Press), 1–19. 10.1016/B978-0-12-815041-2.00001-3

[B25] Dumètre A. Dubey J. P. Ferguson D. J. P. Bongrand P. Azas N. Puech P.-H. (2013). Mechanics of the Toxoplasma gondii oocyst wall. Proc. Natl. Acad. Sci. U. S. A. 110, 11535–11540. 10.1073/pnas.1308425110 23798399 PMC3710823

[B26] Ehret T. Spork S. Dieterich C. Lucius R. Heitlinger E. (2017). Dual RNA-seq reveals no plastic transcriptional response of the coccidian parasite Eimeria falciformis to host immune defenses. BMC Genomics 18, 686. 10.1186/s12864-017-4095-6 28870168 PMC5584376

[B27] Entzeroth R. Mattig F. R. Werner-Meier R. (1998). Structure and function of the parasitophorous vacuole in Eimeria species. Int. J. Parasitol. 28, 1015–1018. 10.1016/S0020-7519(98)00079-4 9724871

[B28] Farhat D. C. Swale C. Dard C. Cannella D. Ortet P. Barakat M. (2020). A MORC-driven transcriptional switch controls Toxoplasma developmental trajectories and sexual commitment. Nat. Microbiol. 5, 570–583. 10.1038/s41564-020-0674-4 32094587 PMC7104380

[B29] Ferguson D. J. P. Dubremetz J.-F. (2020). “Chapter 2 - The ultrastructure of Toxoplasma gondii,” in Toxoplasma gondii (Third Edition). Eds. Weiss L. M. Kim K. (Academic Press), 21–61. 10.1016/B978-0-12-815041-2.00002-5

[B30] Fischer G. J. Keller N. P. (2016). Production of cross-kingdom oxylipins by pathogenic fungi: An update on their role in development and pathogenicity. J. Microbiol. Seoul Korea 54, 254–264. 10.1007/s12275-016-5620-z PMC5107414 26920885

[B31] Gajria B. Bahl A. Brestelli J. Dommer J. Fischer S. Gao X. (2008). ToxoDB: an integrated Toxoplasma gondii database resource. Nucleic Acids Res. 36, D553–D556. 10.1093/nar/gkm981 18003657 PMC2238934

[B32] García-Lunar P. Regidor-Cerrillo J. Ortega-Mora L. M. Gutiérrez-Expósito J. Álvarez-García E (2014). Proteomics reveals differences in protein abundance and highly similar antigenic profiles between Besnoitia besnoiti and Besnoitia tarandi. Vet. Parasitol. 205, 434–443. 10.1016/j.vetpar.2014.09.003 25260331

[B33] García-Sánchez M. Jiménez-Pelayo L. Horcajo P. Regidor-Cerrillo J. Collantes-Fernández E. Ortega-Mora L. M. (2019). Gene Expression Profiling of Neospora caninum in Bovine Macrophages Reveals Differences Between Isolates Associated With Key Parasite Functions. Front. Cell. Infect. Microbiol. 9, 354. 10.3389/fcimb.2019.00354 31681630 PMC6803445

[B34] Gatie M. I. Kelly G. M. (2018). Metabolic profile and differentiation potential of extraembryonic endoderm-like cells. Cell Death Discov. 4, 42. 10.1038/s41420-018-0102-1 PMC6158286 30302276

[B35] Gold D. A. Kaplan A. D. Lis A. Bett G. C. L Rosowski E. E. Cirelli K. M. (2015). The Toxoplasma Dense Granule Proteins GRA17 and GRA23 Mediate the Movement of Small Molecules between the Host and the Parasitophorous Vacuole. Cell Host Microbe 17, 642–652. 10.1016/j.chom.2015.04.003 25974303 PMC4435723

[B36] Guinane C. M. Cotter P. D. (2013). Role of the gut microbiota in health and chronic gastrointestinal disease: understanding a hidden metabolic organ. Ther. Adv. Gastroenterol. 6, 295–308. 10.1177/1756283X13482996 PMC3667473 23814609

[B37] Gupta N. Hartmann A. Lucius R. Voelker D. R (2012). The obligate intracellular parasite Toxoplasma gondii secretes a soluble phosphatidylserine decarboxylase. J. Biol. Chem. 287, 22938–22947. 10.1074/jbc.M112.373639 22563079 PMC3391113

[B38] Hehl A. B. Basso W. U. Lippuner C. Ramakrishnan C. Okoniewski M. Walker R. A. (2015). Asexual expansion of Toxoplasma gondii merozoites is distinct from tachyzoites and entails expression of non-overlapping gene families to attach, invade, and replicate within feline enterocytes. BMC Genomics 16, 66. 10.1186/s12864-015-1225-x 25757795 PMC4340605

[B39] Heitlinger E. Spork S. Lucius R. Dieterich C (2014). The genome of Eimeria falciformis - reduction and specialization in a single host apicomplexan parasite. BMC Genomics 15, 696. 10.1186/1471-2164-15-696 25142335 PMC4287421

[B40] Heo I. Dutta D. Schaefer D. A. Iakobachvili N. Artegiani B. Sachs N. (2018). Modelling Cryptosporidium infection in human small intestinal and lung organoids. Nat. Microbiol. 3, 814–823. 10.1038/s41564-018-0177-8 29946163 PMC6027984

[B41] Hong D.-P. Radke J.-B. White M.-W (2017). Opposing transcriptional mechanisms regulate Toxoplasma development. mSphere 2, e00347–e00316. 10.1128/mSphere.00347-16 28251183 PMC5322347

[B42] Horcajo P. Xia D. Randle N. Collantes-Fernández E. Wastling J. Ortega-Mora L. M. (2018). Integrative transcriptome and proteome analyses define marked differences between Neospora caninum isolates throughout the tachyzoite lytic cycle. Proteomics Infect. Dis. 180, 108–119. 10.1016/j.jprot.2017.11.007 29154927

[B43] Hu D. Wang C. Wang S. Tang X. Duan C. Zhang S. (2018). Comparative transcriptome analysis of Eimeria maxima (Apicomplexa: Eimeriidae) suggests DNA replication activities correlating with its fecundity. BMC Genomics 19, 699. 10.1186/s12864-018-5090-2 30249186 PMC6154952

[B44] Hu D. Tang X. Ben Mamoun C. Wang C. Wang S. Gu X. (2020). Efficient Single-Gene and Gene Family Editing in the Apicomplexan Parasite Eimeria tenella Using CRISPR-Cas9. Front. Bioeng. Biotechnol. 8. 10.3389/fbioe.2020.00128 PMC7052334 32158750

[B45] Jarquín-Díaz V. H. Balard A. Mácová A. Jost J. Roth von Szepesbéla T. Berktold K. (2020). Generalist Eimeria species in rodents: Multilocus analyses indicate inadequate resolution of established markers. Ecol. Evol. 10, 1378–1389. 10.1002/ece3.5992 32076521 PMC7029063

[B46] Jeninga M. D. Quinn J. E. Petter M. (2019). ApiAP2 Transcription Factors in Apicomplexan Parasites. Pathogens 8. 10.3390/pathogens8020047 PMC6631176 30959972

[B47] Jiménez-Meléndez A. Ramakrishnan C. Hehl A. B. Russo G. Álvarez-García E (2020). RNA-Seq Analyses Reveal That Endothelial Activation and Fibrosis Are Induced Early and Progressively by Besnoitia besnoiti Host Cell Invasion and Proliferation. Front. Cell. Infect. Microbiol. 10, 218. 10.3389/fcimb.2020.00218 32500038 PMC7242738

[B48] Jonscher E. Erdbeer A. Günther M. Kurth M. (2015). Two COWP-like cysteine rich proteins from Eimeria nieschulzi (coccidia, apicomplexa) are expressed during sporulation and involved in the sporocyst wall formation. Parasitol. Vectors 8, 395–395. 10.1186/s13071-015-0982-3 PMC4514997 26209229

[B49] Kim W. H. Chaudhari A. A. Lillehoj H. S. (2019). Involvement of T Cell Immunity in Avian Coccidiosis. Front. Immunol. 10. 10.3389/fimmu.2019.02732 PMC6886378 31824509

[B50] Kim K. Jeffers V. Sullivan W. J (2020). “Chapter 21 - Regulation of gene expression inToxoplasma gondii,” in Toxoplasma gondii (Third Edition), eds. L. M. Weiss and K. Kim (Cambridge, Massachusetts: Academic Press), 941–982. 10.1016/B978-0-12-815041-2.00021-9

[B51] Kogut M. H. (1990). “Host specificity of the coccidia,” in Coccidiosis Man Domestic Animals, (Boca Raton, Florida: CRC Press, Inc) vol. 1, 44–55.

[B52] Krishnan A. Kloehn J. Lunghi M. Chiappino-Pepe A. Waldman B. S. Nicolas D. (2020). Functional and Computational Genomics Reveal Unprecedented Flexibility in Stage-Specific Toxoplasma Metabolism. Cell Host Microbe 27, 290–306.e11. 10.1016/j.chom.2020.01.002 31991093

[B53] Kvičerová J. Hypša V. (2013). Host-Parasite Incongruences in Rodent Eimeria Suggest Significant Role of Adaptation Rather than Cophylogeny in Maintenance of Host Specificity. PLoS One 8, e63601. 10.1371/journal.pone.0063601 23861732 PMC3701668

[B54] Lal K. Bromley E. Oakes R. Prieto J. H. Sanderson S. J. Kurian D. (2009). Proteomic comparison of four Eimeria tenella life cycle stages: unsporulated oocyst, sporulated oocyst, sporozoite and second- generation merozoite. Proteomics 9, 4566–4576. 10.1002/pmic.200900305 19795439 PMC2947549

[B55] Ma D. Huang Y. Ma C. Zhang L. Wang J. Wang D. (2019). Eimeria tenella: specific EtAMA1-binding peptides inhibit sporozoite entry into host cells. Poult. Sci. 98, 4480–4491. 10.3382/ps/pez298 31149727

[B56] Mácová A. Hoblíková A. Hypša V. Stanko M. Martinů J. Kvičerová K. (2018). Mysteries of host switching: Diversification and host specificity in rodent-coccidia associations. Mol. Phylogenet. Evol. 127, 179–189. 10.1016/j.ympev.2018.05.009 29753710

[B57] Mai K. Sharman P. A. Walker R. A. Katrib M. Souza D. D. McConville M. J. (2009). Oocyst wall formation and composition in coccidian parasites. Mem. Inst. Oswaldo Cruz 104, 281–289. 10.1590/S0074-02762009000200022 19430654

[B58] Martorelli Di Genova B. Wilson S. K. Dubey J. P. Knoll L. J. (2019). Intestinal delta-6-desaturase activity determines host range for Toxoplasma sexual reproduction. PLoS Biol. 17, e3000364. 10.1371/journal.pbio.3000364 31430281 PMC6701743

[B59] Mayoral J. Di Cristina M. Carruthers V. B. Weiss L. M. (2020). “Toxoplasma gondii: BradyzoiteDifferentiation In Vitro and In Vivo,” in Toxoplasma gondii (New York, NY: Springer US), 269–282. 10.1007/978-1-4939-9857-9_15 PMC7059825 31758458

[B60] McGraw T. E. Mittal V. (2010). Metabolism regulates differentiation. Nat. Chem. Biol. 6, 176–177. 10.1038/nchembio.324 20154665

[B61] Metsalu T. Vilo J. (2015). ClustVis: a web tool for visualizing clustering of multivariate data using Principal Component Analysis and heatmap. Nucleic Acids Res. 43, W566–W570. 10.1093/nar/gkv468 25969447 PMC4489295

[B62] Montes C. Rojo F. Hidalgo R. Ferre I. Badiola C (1998). Selection and development of a Spanish precocious strain of Eimeria necatrix. Vet. Parasitol. 78, 169–183. 10.1016/S0304-4017(98)00125-3 9760059

[B63] Must K. Hytönen M. K. Orro T. Lohi H. Jokelainen P (2017). Toxoplasma gondii seroprevalence varies by cat breed. PLoS One 12. 10.1371/journal.pone.0184659 PMC5590984 28886182

[B64] Naruzawa E. S. Malagnac F. Bernier L (2015). Effect of linoleic acid on reproduction and yeast–mycelium dimorphism in the Dutch elm disease pathogens. Botany 94, 31–39. 10.1139/cjb-2015-0156

[B65] Noack S. Chapman H. D. Selzer P. M (2019). Anticoccidial drugs of the livestock industry. Parasitol. Res. 118, 2009–2026. 10.1007/s00436-019-06343-5 31152233 PMC6611755

[B66] Obukowicz M. G. Raz A Pyla P. D. Rico J. G. Wendling J. M. Needleman P (1998). Identification and Characterization of a Novel Δ6/Δ5 Fatty Acid Desaturase Inhibitor As a Potential Anti-Inflammatory Agent. Biochem. Pharmacol. 55, 1045–1058. 10.1016/S0006-2952(97)00665-5 9605428

[B67] Olson W. J. Martorelli Di Genova B. Gallego-Lopez G. Dawson A. R. Stevenson D. Amador-Noguez D. (2020). Dual metabolomic profiling uncovers Toxoplasma manipulation of the host metabolome and the discovery of a novel parasite metabolic capability. PLoS Pathog. 16, e1008432. 10.1371/journal.ppat.1008432 32255806 PMC7164669

[B68] Ong Y.-C. Boyle J. P. Boothroyd J. C (2011). Strain-Dependent Host Transcriptional Responses to Toxoplasma Infection Are Largely Conserved in Mammalian and Avian Hosts. PLoS One 6, e26369. 10.1371/journal.pone.0026369 22022607 PMC3192797

[B69] Pakandl M. (2005). Selection of a precocious line of the rabbit coccidium Eimeria flavescens Marotel and Guilhon (1941) and characterisation of its endogenous cycle. Parasitol. Res. 97, 150–155. 10.1007/s00436-005-1411-x 15986244

[B70] Pernas L. Boothroyd J. C. (2010). Association of host mitochondria with the parasitophorous vacuole during Toxoplasma infection is not dependent on rhoptry proteins ROP2/8. Int. J. Parasitol. 40, 1367–1371. 10.1016/j.ijpara.2010.07.002 20637758 PMC2939271

[B71] Pfefferkorn L. C. Pfefferkorn E. R. (1980). Toxoplasma gondii: Genetic recombination between drug resistant mutants. Exp. Parasitol. 50, 305–316. 10.1016/0014-4894(80)90034-X 6448752

[B72] Poran A. Nötzel C. Aly O. Mencia-Trinchant N. Harris C. T. Guzman M. L. (2017). Single-cell RNA sequencing reveals a signature of sexual commitment in malaria parasites. Nature 551, 95–99. 10.1038/nature24280 29094698 PMC6055935

[B73] Possenti A. Cherchi S. Bertuccini L. Pozio E. Dubey J. P. Spano F (2010). Molecular characterisation of a novel family of cysteine-rich proteins of Toxoplasma gondii and ultrastructural evidence of oocyst wall localisation. Int. J. Parasitol. 40, 1639–1649. 10.1016/j.ijpara.2010.06.009 20708619

[B74] Quiroz-Castañeda R. E. Dantán-González E. (2015).Control of Avian Coccidiosis: Future and Present Natural Alternatives. Available at: https://www.hindawi.com/journals/bmri/2015/430610/ (Accessed 01-Apr-2020). 10.1155/2015/430610 PMC4346696 25785269

[B75] Radke J. B. Lucas O. De Silva E. K. Ma Y. Sullivan W. J. Weiss L. M. (2013). ApiAP2 transcription factor restricts development of the Toxoplasma tissue cyst. Proc. Natl. Acad. Sci. 110, 6871–6876. 10.1073/pnas.1300059110 23572590 PMC3637731

[B76] Ramakrishnan C. Walker R. A. Eichenberger R. M. Hehl A. B. Smith N. C (2017). The merozoite-specific protein, TgGRA11B, identified as a component of the Toxoplasma gondii parasitophorous vacuole in a tachyzoite expression model. Int. J. Parasitol. 47, 597–600. 10.1016/j.ijpara.2017.04.001 28526607

[B77] Ramakrishnan C. Maier S. Walker R. A. Rehrauer H. Joekel D. E. Winiger R. R. (2019). An experimental genetically attenuated live vaccine to prevent transmission of Toxoplasma gondii by cats. Sci. Rep. 9, 1–14. 10.1038/s41598-018-37671-8 30728393 PMC6365665

[B78] Reid A. J. Vermont S. J. Cotton J. A. Harris D. Hill-Cawthorne G. A. Könen-Waisman S. (2012). Comparative Genomics of the Apicomplexan Parasites Toxoplasma gondii and Neospora caninum: Coccidia Differing in Host Range and Transmission Strategy. PLoS Pathog. 8, e1002567. 10.1371/journal.ppat.1002567 22457617 PMC3310773

[B79] Reid A. J. Blake D. P. Ansari H. R. Billington K. Browne H. P. Bryant J. (2014). Genomic analysis of the causative agents of coccidiosis in domestic chickens. Genome Res. 24, 1676–1685. 10.1101/gr.168955.113 25015382 PMC4199364

[B80] Rentschler L. A. Hirschberger L. L. Stipanuk M. H. (1986). Response of the kitten to dietary taurine depletion: Effects on renal reabsorption, bile acid conjugation and activities of enzymes involved in taurine synthesis. Comp. Biochem. Physiol. Part B Comp. Biochem. 84, 319–325. 10.1016/0305-0491(86)90084-2 3743026

[B81] Robben P. M. Sibley L. D. (2004). Food- and waterborne pathogens: you are (infected by) what you eat! Microbes Infect. 6, 406–413. 10.1016/j.micinf.2003.12.016 15101398

[B82] Russell D. G. Sinden R. E. (1981). The role of the cytoskeleton in the motility of coccidian sporozoites. J. Cell Sci. 50, 345–359. 7033252 10.1242/jcs.50.1.345

[B83] Schneiders G. H. Foutz J. C. Milfort M. C. Ghareeb A. F. A. Fuller A. L. Rekaya R. (2020). Heat stress reduces sexual development and affects pathogenesis of Eimeria maxima in meat-type chickens. Sci. Rep. 10, 10736. 10.1038/s41598-020-67330-w 32612102 PMC7329875

[B84] Schwarz J. A. Fouts A. E. Cummings C. A. Ferguson D. J. P. Boothroyd J. C (2005). A novel rhoptry protein in Toxoplasma gondii bradyzoites and merozoites. Mol. Biochem. Parasitol. 144, 159–166. 10.1016/j.molbiopara.2005.08.011 16182390

[B85] Shahbazi G. Rad N. H. Madani R. Matin S. Mortazavi P. Jangjou A. H. (2019). Toxoplasma gondii in Aborted Fetuses of Sheepin Ardebil Area, North-West of Iran. Iran. J. Parasitol. 14, 430–435. 10.18502/ijpa.v14i3.1482 31673261 PMC6815854

[B86] Sheffield H. G. (1970). Schizogony in Toxoplasma gondii: an electron microscope study. Proc. Helminthol. Soc Wash. 37, 237–242.

[B87] Sinclair A. J. McLean J. G. Monger E. A (1979). Metabolism of linoleic acid in the cat. Lipids 14, 932–936. 10.1007/BF02533508 513981

[B88] Sivajothi S. Reddy B. S. Rayulu V. C (2016). Study on impression smears of hepatic coccidiosis in rabbits. J. Parasitol. Dis. Off. Organ Indian Soc Parasitol. 40, 906–909. 10.1007/s12639-014-0602-8 PMC4996215 27605807

[B89] Smith T. G. Walliker D. Ranford-Cartwright L. C. (2002). Sexual differentiation and sex determination in the Apicomplexa. Trends Parasitol. 18, 315–323. 10.1016/S1471-4922(02)02292-4 12379952

[B90] Sokol S. L. Primack A. S. Nair S. C. Wong Z. S. Tembo M. Verma S. K. (2018). Dissection of the in vitro developmental program of Hammondia hammondi reveals a link between stress sensitivity and life cycle flexibility in Toxoplasma gondii. eLife 7, e36491. 10.7554/eLife.36491 29785929 PMC5963921

[B91] Sokol S. L. Wong Z. S. Boyle J. P. Dubey J. P. (2020). Generation of Toxoplasma gondii and Hammondia hammondi Oocysts and Purification of Their Sporozoites for Downstream Manipulation. Methods Mol. Biol. Clifton NJ 2071, 81–98. 10.1007/978-1-4939-9857-9_4 PMC7646208 31758447

[B92] Striepen B. Jordan C. N. Reiff S. van Dooren G. G (2007). Building the Perfect Parasite: Cell Division in Apicomplexa. PLoS Pathog. 3, e78. 10.1371/journal.ppat.0030078 17604449 PMC1904476

[B93] Strout R. G. Solis J. Smith S. C. Dunlop W. R (1965). In vitro cultivation of Eimeria acervulina (Coccidia). Exp. Parasitol. 17, 241–246. 10.1016/0014-4894(65)90064-0 5856846

[B94] Su S. Hou Z. Liu D. Jia C. Wang L. Xu J. (2017). Comparative transcriptome analysis of second-and third-generation merozoites of Eimeria necatrix. Parasitol. Vectors 10, 388. 10.1186/s13071-017-2325-z PMC5559809 28814335

[B95] Taubert A. Silva L. M. R. Velásquez Z. D. Larrazabal C. Lütjohann D. Hermosilla C (2018). Modulation of cholesterol-related sterols during Eimeria bovis macromeront formation and impact of selected oxysterols on parasite development. Mol. Biochem. Parasitol. 223, 1–12. 10.1016/j.molbiopara.2018.06.002 29909067

[B96] Templeton T. J. Lancto C. A. Vigdorovich V. Liu C. London N. R. Hadsall K. Z. (2004). The Cryptosporidium oocyst wall protein is a member of a multigene family and has a homolog in Toxoplasma. Infect. Immun. 72, 980–987. 10.1128/IAI.72.2.980-987.2004 14742544 PMC321576

[B97] Vega-Rodriguez J. Perez-Barreto D. Ruiz-Reyes A. Jacobs-Lorena M (2015). Targeting molecular interactions essential for Plasmodium sexual reproduction. Cell. Microbiol. 17, 1594–1604. 10.1111/cmi.12458 25944054 PMC4668941

[B98] Vrba V. Pakandl M. (2015). Host specificity of turkey and chicken Eimeria: controlled cross-transmission studies and a phylogenetic view. Vet. Parasitol. 208, 118–124. 10.1016/j.vetpar.2015.01.017 25660426

[B99] Wainwright K. E. Miller M. A. Barr B. C. Gardner I. A. Melli A. C. Essert T. (2007). Chemical inactivation of Toxoplasma gondii oocysts in water. J. Parasitol. 93, 925–931. 10.1645/GE-1063R.1 17918377

[B100] Waldman B. S. Schwarz D. Wadsworth M. H. Saeij B. S. Shalek A. K. Lourido S (2020). Identification of a Master Regulator of Differentiation in Toxoplasma. Cell 180, 359–372.e16. 10.1016/j.cell.2019.12.013 31955846 PMC6978799

[B101] Walker R. A. Ferguson D. J. P. Miller C. M. D. Smith N. C (2013). Sex and Eimeria: a molecular perspective. Parasitology 140, 1701–1717. 10.1017/S0031182013000838 23953058

[B102] Walker R. A. Sharman P. A. Miller C. M. Lippuner C. Okoniewski M. Eichenberger R. M. (2015). RNA Seq analysis of the Eimeria tenella gametocyte transcriptome reveals clues about the molecular basis for sexual reproduction and oocyst biogenesis. BMC Genomics 16, 94. 10.1186/s12864-015-1298-6 25765081 PMC4345034

[B103] Wang Z.-D. Liu H. H. Ma Z. X. Ma H. Y. Li Z. Y. Yang Z. B. (2017). Toxoplasma gondii Infection in Immunocompromised Patients: A Systematic Review and Meta-Analysis. Front. Microbiol. 8, 389–389. 10.3389/fmicb.2017.00389 28337191 PMC5343064

[B104] Wang Z. T. Verma S. K. Dubey J. P. Sibley L. D (2017). The aromatic amino acid hydroxylase genes AAH1 and AAH2 in Toxoplasma gondii contribute to transmission in the cat. PLoS Pathog. 13, e1006272. 10.1371/journal.ppat.1006272 28288194 PMC5363998

[B105] Ware M. W. Augustine S. A. J. Erisman D. O. See M. J. Wymer L. Hayes S. L. (2010). Determining UV Inactivation of Toxoplasma gondii Oocysts by Using Cell Culture and a Mouse Bioassay. Appl. Environ. Microbiol. 76, 5140–5147. 10.1128/AEM.00153-10 20543052 PMC2916465

[B106] Wilke G. Funkhouser-Jones L. J. Wang Y. Ravindran S. Wang Q. Beatty W. L. (2019). A Stem-Cell-Derived Platform Enables Complete Cryptosporidium Development In Vitro and Genetic Tractability. Cell Host Microbe 26, 123–134.e8. 10.1016/j.chom.2019.05.007 31231046 PMC6617391

[B107] Zhou C.-X. Zhu X.-Q. Elsheikha H. M. He S. Li Q. Zhou D.-H. (2016). Global iTRAQ-based proteomic profiling of Toxoplasma gondii oocysts during sporulation. J. Proteomics 148, 12–19. 10.1016/j.jprot.2016.07.010 27422377

